# Off-label use of medicines in South Africa: a review

**DOI:** 10.1186/s13023-024-03476-4

**Published:** 2024-11-29

**Authors:** N. N. Ngcobo, L. J. Mathibe

**Affiliations:** 1 Discipline of Pharmaceutical Sciences, School of Health Sciences, University of KwaZulu-Natal, Durban, South Africa; 2 Division of Pharmacology (Therapeutics), Discipline of Pharmaceutical Sciences, Nelson R. Mandela School of Medicine, University of KwaZulu-Natal, Durban, South Africa

**Keywords:** Off-label, Regulations, FDA, SAHPRA, Evidence-based medicine, Rare diseases

## Abstract

**Background:**

Off-label use of medicinal products has become an important part of mainstream and legitimate medical practice worldwide. This practice is common in oncology, obstetrics, paediatrics, and in the management of infectious diseases (notably HIV), and inflammatory conditions as well as in rare and/or orphan diseases. However, the off-label use of medicines recently-raised many clinical and legal difficulties, not only among medical practitioners but also among pharmacists and other healthcare professionals.

**Aim:**

This paper, therefore, highlights the advantages (such as cost saving for both the patient and the country/insurance that is paying for the medication) and disadvantages (insufficient evidence available) of the use of medicines to treat specific conditions or indications for which they are currently not registered.

**Conclusion:**

Off-label drug use can be likened to a double-edged sword, offering valuable opportunities for medical practitioners while carrying potential risks. When the scientific basis for off-label use is unclear, it may place patients at risk of unapproved experimentation, unforeseen health hazards, and ineffective treatments. Hence, there is a pressing need in South Africa for clear regulatory guidelines on off-label drug use. Additionally, the timely review and approval of new indications for medicines, backed by robust scientific evidence, are essential. This would reduce the significant burden and inherent risks faced by medical practitioners when using medicines off-label to provide compassionate care.

## Introduction

In South Africa, the Medicines Act 101 of 1965, as amended, gives the South African Health Products Regulatory Authority (SAHPRA) the powers to assess the scientific evidence, often provided by the applicant, and to decide whether to register medicines or not [1]. Usually the criteria which SAHPRA uses, include evidence on; (i) the effectiveness (or efficacy) of medicine for management of specific indication(s), (ii) safety profile in the specific population for which registration is sought, and (iii) the quality of the health products to be administered without further manipulations [2]. Similar criteria are used globally by other similar regulatory bodies such as the Food and Drug Administration (FDA) in the United States of America (USA) as well as the Therapeutics Goods Administration (TGA) in Australia.

However, medicines such as amitriptyline are often prescribed on an off-label basis. This is also known as the off-label drug use (OLDU) [3]. Meaning, such medicines are prescribed and utilised for additional indications for which regulatory authorities have not registered or approved. [4, 5] The OLDU has become an important part of mainstream and legitimate medical practice worldwide. It is commonly-practiced in disciplines and specialties such as oncology, obstetrics, paediatrics, infectious diseases notably human immunodeficiency virus (HIV), pain and inflammatory conditions as well as in rare and/or orphan diseases [4]. In numerous cases of perinatally HIV-1 infected children, OLDU, has been an optimal strategy to prevent immunological and clinical deterioration in the early start of highly active antiretroviral therapy (HAART) [6].

However, while OLDU use can provide necessary treatment options in certain situations, it is fraught with potential risks and challenges, not only among medical practitioners but also for pharmacists and other healthcare professionals. Clinicians must weigh these risks carefully, rely on the best available evidence, and ensure transparent communication with patients and/or caregivers regarding the off-label use of medications. Therefore, in this narrative review, scientific evidence regarding the advantages and disadvantages of prescribing and utilising medicines, to treat diseases for which they are not registered, is synthesised to make the vast body of knowledge comprehensible to healthcare providers and regulatory authorities.

### Regulatory framework

While off-label and unlicensed practices are well-characterized in several developed countries and regions, there are concerns about the limited number of studies addressing these practices in developing countries, including South Africa [7, 8]. Recent reviews indicate that unlicensed use of medicines can account for up to 75% of medication use among hospitalized children in some studies [9]. However, Oshikoya et al., reported that off-label prescriptions were only 7.7% among children with chronic diseases attending specialty paediatric clinics in Nigeria [10]. Despite this, earlier studies highlighted a higher risk of drug-drug interactions among paediatric patients [11]. Additionally, there were significant concerns regarding the off-label use of pentazocine among paediatric surgical patients in Nigeria, with most children experiencing between two and seven adverse event [12].

Developing countries, including South Africa, are particularly affected by off-label and unlicensed medicine use. This is due to the significant proportion of the population aged 0 to 18 years and their higher susceptibility to infectious diseases [13–15]. For example, Southern Africa has a high incidence of children born to mothers with HIV, which contrasts with the situation in higher-income countries [16].

Regulations regarding OLDU vary across countries. In several European countries, including the UK, special statutory regulations for OLDU have been partly omitted, similar to Germany [17]. In the UK, drug prescribers receive guidance from the General Medical Council on off-label prescribing practices stipulating that clinicians must be convinced of the drug’s effectiveness and safety for off-label use, supported by sufficient evidence or experience [18]. Conversely, certain countries have implemented specific statutory regulations. For example, Article 9 of the Swiss Federal Law on Medicinal Products and Medical Devices allows a temporary regulatory exemption for the use of non-approved drugs if they are intended to treat life-threatening diseases, are expected to offer significant therapeutic benefits while ensuring health protection, and if no comparable medicinal product is available [19]. Moreover, Article 26 of the same law mandates that clinicians adhere to established medical and pharmaceutical science standards when prescribing such drugs [19]. This requires a specialist to substantiate the medical indication with a thorough diagnosis [17]. A think-tank, which includes Swissmedic, hospital pharmacists, and canton representatives, formalized these medical and pharmaceutical care obligations in a report [17]. When these guidelines are followed, off-label use is legally permitted as an exception, despite the general authorization requirements [17]. In Spain, off-label prescribing must be exceptional, limited to cases where no authorized alternatives are available for the patient, and it requires patient consent [18]. In the Netherlands, off-label prescriptions are permitted only if relevant professional bodies have established protocols or professional standards for that specific use [18].

Studies have reported that 17% to 40% of prescription drugs in ambulatory care are prescribed for conditions that are not approved by the FDA or other regulatory agencies, meaning they are used for indications that have not received official approval [20, 21]. Oberoi (2015), reported that the extent of OLDU varies based on factors such as the level of healthcare available, the specific subspecialty involved, and certain patient characteristics [22]. The study found that OLDU in several European countries ranged from 10.8% to 66.0% [22]. In the United States, about one-third of antibiotic prescriptions are linked to inappropriate use, which has played a significant role in the increase of antibiotic resistance [23].

A European Union report indicated that over 55% of clinicians engage in off-label prescribing [24]. In the United States, Bradford et al., estimate that off-label prescriptions account for 29–38% based on data from the National Ambulatory Medical Care Survey [25]. For cancer medications, off-label usage rates are estimated at 17–20% [21]. In France, Tunçel (2019) estimated that 21% of prescriptions for depression drugs were for off-label uses [26]. In India, the Drug Controller General of India (DCGI) is the regulatory authority responsible for approving new drugs. However, there are currently no clear guidelines on the off-label use of drugs [22, 27]. Under Indian law, prescribing drugs for unapproved indications is not permitted. Amendments to the Indian Medical Council Act made off-label prescribing illegal [22], but, recently OLDU is being practiced in India even though there are no stringent guidelines [28, 29]. Additionally, off-label marketing by pharmaceutical companies is considered a legal violation and is an offense under the Drug and Magic Remedies (Objectionable Advertisements) Act, 1954 [22, 28, 29].

### Medicines commonly-prescribed on an off-label basis

There is considerable evidence regarding medical practitioners’ regular reliance on OLDU. This practice is to a greater extent applied in the non-traditional management approaches for pain, mental disorders and cardiovascular diseases among others.

#### Gabapentin

The FDA approved gabapentin’s clinical use in 1994 as an adjunct therapy for the treatment of partial seizures in adults with epilepsy [30]. Gabapentin reduces neuronal excitability in patients with epilepsy. However, it is often prescribed for the management of neuropathic pain (ICD-10: M79. 2), bipolar disorder (ICD-10: F31) [31] and migraine (ICD-10: G43. 909) [32, 33] Wiffen et al. [34], reported gabapentin being effective in pain relief for more than 50% of patients treated for postherpetic neuralgia and peripheral diabetic neuropathy at doses of 1 800 mg to 3 600 mg daily. Recently, Tubog et al. [35] reported that gabapentin is effective in the management of postoperative pain in the first 24 h after surgery. However, there is a need for randomised clinical trials to assess the effectiveness of gabapentin in the management of chemotherapy-induced neuropathy.

#### Carbamazepine

Carbamazepine regulates the voltage-gated sodium channel which results in an inhibition of action potential and reduced synaptic transmission [36]. The FDA has approved for acute manic and mixed episodes in bipolar I disorder (ICD- 10: F31. 2) [37]. Carbamazepine is used on an off-label basis for refractory schizophrenia (ICD- 10: F20. 5) [38], restless leg syndrome (ICD- 10: G25. 81) [39] and severe alcohol withdrawal syndrome (ICD- 10: F10. 239) [40] among many others. Winkelmann et al. [39], reported carbamazepine as one of the pharmacological agents that improve both positive and negative symptoms in schizophrenic patients. Leucht et al. [38], reported that carbamazepine promotes an additional response in patients with schizophrenia in many cases where therapeutic effect(s) are not evident with treatment with antipsychotic agents. As a result, carbamazepine is often used on an off-label basis for maintenance therapy for bipolar disorder. However, it has not been shown to prevent seizures associated with alcohol withdrawal when compared to benzodiazepines [40]. Recently, a study by Latifi and Messer [41], demonstrated clinical efficacy in the treatment of patients with moderate to severe alcohol withdrawal syndrome. Carbamazepine has been reported to be a tolerable and effective pharmacological agent for the treatment of restless leg syndrome [39, 42]. The treatment of neuropathic pain and fibromyalgia is another prominent off-label use of carbamazepine [34]. Saeed et al. [43] suggested that carbamazepine has clinically significant pain relief (in patients with diabetic neuropathy) with improved quality of life and good tolerability. Carbamazepine has been reported the efficacy of carbamazepine in the treatment of agitation and aggression associated with dementia [39, 44]. However, the response to treatment is patient-dependent (patient-tailored treatment).

#### Imipramine and fluoxetine

Tertiary amine tricyclic antidepressants such as imipramine prevent norepinephrine and serotonin from being reabsorbed. [30] Although imipramine is FDA approved for the treatment of depression (ICD- 10: F32. 9), it is now used on an off-label basis for the treatment of nocturnal enuresis (ICD- 10: F98. 02), panic disorder (ICD- 10: F41. 0), and neuropathic pain (ICD-10: M79. 2) to mention a few [30]. Imipramine is a treatment option for children over the age of six who suffer from nocturnal enuresis [45]. A study by Seyfhashemi et al. [46], suggested, imipramine can be used to prevent urinary incontinence by its ability to enhance the contractile effects of noradrenaline urethral smooth muscles. Fayez and Gupta [47], suggested, imipramine can also be used to treat panic disorder. Hearn et al. [48], suggested the use of imipramine in treating chronic neuropathic pain with starting dosages between 10 and 25 mg daily, increasing to 75 mg daily if necessary.

Fluoxetine has been approved by the FDA for its significant effect in the management of major depressive disorder (ICD- 10: F33. 1) and obsessive–compulsive disorder (ICD- 10: F42. 9) [49]. Unregistered uses for fluoxetine include social anxiety disorder (ICD- 10: F40. 10) [50], post-traumatic disorder (ICD- 10: F43.1) in adults [51] borderline personality disorder (ICD- 10: F60. 3) [52], and Raynaud phenomenon (ICD- 10: I73. 0) [53]. Martin et al., reported the use of fluoxetine at a dosage of 20–80 mg is effective in the management of post-traumatic disorder [54]. Fluoxetine has been reported to efficiently and effectively treat and prevent the relapse of PTSD symptoms [54]. Sohel et al. [50], confirm the tolerability of fluoxetine (20 mg daily) and suggest that it would be effective as a novel treatment for Raynaud’s phenomenon. Interestingly, Li et al. [55], strongly suggested that the use of fluoxetine by males can counteract the contraction of the vas deferens, thereby, delaying ejaculation i.e. premature ejaculation.

#### Propranolol and timolol

Β-adrenoreceptor antagonists, such as propranolol are registered for the treatment of cardiovascular failure (ICD- 10: I50. 9), atrial fibrillation (ICD- 10: I48.91), and coronary artery disease (ICD- 10: I25. 41) [56]. Propranolol can enhance the sympathetic response in angina and tachyarrhythmias while preventing acute ischemic attacks [56]. This it has been demonstrated to improve mortality and morbidity in individuals with hypertension complicated by heart failure, angina, or any history of previous myocardial infarctions [56]. Not only does propranolol play a significant role in the treatment of cardiovascular disease, but it also has several implications for the treatment of processes that are not related to cardiovascular disease, such as the treatment of performance anxiety (ICD- 10: F41. 9) [56]. Shahrokhi and Gupta (2023), reported the use of propranolol at doses 40–120 mg a day in the treatment for restless leg syndrome [56, 57]. Propranolol (40–320 mg daily) can also be used for migraine prophylaxis, therapeutic benefits may take up to 12 weeks to become apparent when using an adequate dose of the medication [58].

Timolol is a nonselective β-blocker that can be taken orally or topically. It is mainly used to diminish intraocular pressure in patients with open-angle glaucoma (ICD- 10: H40. 11) and ocular hypertension (ICD- 10: H40. 05) [59]. Timolol has likewise been displayed to successfully treat and limit slight, superficial juvenile hemangiomas (ICD- 10: D18. 0) [60]. Systemic use of timolol can be an important additional agent in the regimen managing hypertension (ICD- 10: R03. 0), myocardial infarction necrosis (ICD- 10: I21. 9), and migraine prophylaxis [61]. A study by Carcel et al. [62], recommended the use of timolol, 10 mg twice daily, for the prophylactic treatment of migraines. In cases where the response is inadequate, the dose can be increased to 30 mg daily [62, 63]. Recently, studies have demonstrated that topical timolol treatment is effective and well-tolerated treatment for superficial infantile hemangiomas [64, 65]. Wiysonge [66], reported timolol as an essential antihypertensive agent. The study showed that an average daily dosage of 15—30 mg will help achieve optimal levels of blood pressure with no significant side effects [61, 66].

#### Atorvastatin

Atorvastatin is a lipid-lowering agent indicated for hypercholesterolaemia (ICD- 10: E78. 00), and prevention of cardiovascular disease [30]. However, atorvastatin has been used on an off-label basis for chronic heart failure (ICD- 10: I50. 22), diabetic retinopathy (ICD- 10: E11. 319), prevention of atrial fibrillation, and rheumatoid arthritis (ICD- 10: M06. 9) [30]. Soulaidopouloset al. [67], reported administration of atorvastatin (40 mg daily for 6 months) suggests that atorvastatin may affect markers for cardiac disease as well as some parameters for clinical inflammatory changes, in patients with rheumatoid arthritis, thus, alleviate disease progression. Hung et al. [68] reported an improvement in atrial fibrillation after the use of atorvastatin. Szyguła-Jurkiewicz et al. [69], reported that administration of atorvastatin in patients with chronic heart failure results in decrease in the frequency of hospitalisation and a reduction in the rates associated with cardiovascular mortality and sudden cardiac death. Recently, a study by Shi et al. [70], suggested a potential use of atorvastatin in diabetic retinopathy. It is believed that early initiation of atorvastatin has advantageous results in an intensive control of blood lipid levels which ultimately delay the development of diabetic retinopathy.

## Advantages of off-label use of medicines

### Prompt utilisation of new treatment options

Off-label prescribing gives medical practitioners the freedom to promptly use new treatment options based on the latest evidence [30]. This means medical practitioners can prescribe, albeit with certain restrictions or conditions, unapproved medicines relying mainly on the available scientific data and appropriate medical practice for certain conditions [30]. For example, isosorbide dinitrate is often used to treat patients with acute strangulated internal haemorrhoids – thereby avoiding the risk of incontinence disorders after conventional surgical treatment [71]. On the other hand, metformin, an effective fertility drug in non-obese women with polycystic ovary syndrome, has several advantages over other first-line treatments for non-ovulatory infertility such as clomiphene [72]. For women who are resistant to clomiphene, metformin alone or in combination with clomiphene is an effective next step [72].

### Financial gains

The financial aspects of OLDU remain arguable. The overall benefit favours the pharmaceutical companies the most, as compared to the end user of the pharmaceutical product [73]. From the medical practitioners’ perspective, by understanding the OLDU and prescriber patterns, it is easy to cut down on costs which in turn would positively affect the overall budget [4]. From a business perspective, it is not very enticing for a pharmaceutical company to apply for additional approval of a new application later in the patent term of the drug. The cost of developing a new use of an old drug can outweigh the benefits of regulatory approval [4]. In the case of generic drugs, innovative drugs are frequently used, especially for innovative drugs that are undergoing expensive clinical trials that may be underfunded and may provide unsubstantiated evidence. If so, this is a risk no one wants to take. In addition, manufacturers can be discouraged by the OLDU from conducting well-controlled clinical trials [9, 22]. From a patient perspective, the OLDU could be cost-saving since generics are being used off-label. This would also mean the insurer/medical aid will pay the lesser price which will lead to significant savings [5].

Oberoi (2015), concluded that permitting the off-label promotion of drugs for untested and unproven benefits prioritizes industry profits over public health [22]. Without evidence of actual benefits, it is impossible to accurately assess the risk–benefit ratio, potentially putting patients at unnecessary risk [22]. Previous research on factors related to economic incentives and the availability of scientific evidence indicates that OLDU tends to be higher when treatment options are limited and when reimbursement policies are less restrictive, which may otherwise discourage off-label prescribing. Regarding welfare effects, Bradford et al. [25] and Tunçel [26] suggest that OLDU can enhance welfare by substituting less expensive off-label drugs for similar on-label drugs. Tunçel [26] estimates that replacing on-label with off-label treatments for depression would lead to a 15% increase in expenditures without significant changes in health outcomes. However, these studies do not account for non-pharmaceutical medical expenses and/or disability in their welfare assessments.

A study by Blankart et al. [21], found that using only on-label drugs, compared to the average level of OLDU in 2015, could result in savings of $515 per individual in healthcare and work-loss costs. Off-label prescriptions account for approximately 21% of the overall use of medications among the 160 most frequently prescribed drugs in the U.S. This significant difference is partly due to the more intensive pharmaceutical promotion that newly approved drugs receive [21]. The study also observed that marketing expenditures dropped by about 50–60% in the years following the introduction of generics [21].

### Sequential application for registration of additional indications

Evidence that emanates from OLDU provides pharmaceutical companies with valuable data to support subsequent applications for approval of additional indication(s) for the use of the same medicine(s) [73]. For example, the FDA originally approved propranolol for the treatment of cardiac arrhythmias in 1968 but approved propranolol for the treatment of hypertension and angina pectoris in 1978, the drug’s very regular therapeutic use [30]. Even more surprisingly, propranolol was found to avert migraine attacks in patients with arrhythmias or angina. After many years of off-label use for migraine, the application was finally approved in 1979 [62].

### New and rare diseases

OLDU is useful in patients with rare diseases and may be the only treatment available [73]. Many anti-cancer drugs that were first approved to treat one type of cancer have been tried to treat another type of cancer [74]. Numerous studies have found that off-label cancer drug use is more prevalent in regions where clinicians are located near clinical trial units. These studies further noted that off-label use increases by 85% when randomized controlled trials demonstrate that an off-label use of a cancer drug improves patient survival [21]. Mitomycin is indicated, for example, for the treatment of gastric and pancreatic cancers. In addition, it meets standard treatments approved for the management of lung, bladder, breast, cervical, and other carcinomas [74]. However, it is not approved by the FDA for these additional indications.

### Populations where medicines are needed but difficult to conduct research

The use of drugs outside of the package insert recommendations for indications, age, route, dose, and frequency is the most common definition of off-label use [4]. Often drugs may not have been studied and approved for a particular population, for example, in paediatric, geriatric or pregnant patients [73]. After numerous studies, a priority list of medicines requiring approval for paediatric use in Australia has been established, and an Australian paediatric prescribing guide may be developed in the coming years as suggested by a study conducted by Ballard et al. [75] The study reported on the off-label use of numerous pharmaceutical agents at one of the hospitals in Australia [75]. However, ongoing efforts are still necessary to enhance paediatric drug testing, registration, and utilization. Relative lack of data can be attributed to many causes, together with unfamiliarity with age-associated in paediatric patients, ethical considerations when conducting paediatric studies [39], and lack of financial incentives for the pharmaceutical industry [4]. For example, the commonly used dexmedetomidine is approved and authorised for sedation via the intravenous (IV) route in adults, but is not indicated for adults and children when administered by the inhalation route [76].

Obstetricians often prescribe medications for indications other than those listed on the product label. [5] Medicines such as nifedipine, isosorbide dinitrate, betamethasone, and aspirin are often used on an off-label basis among pregnant patients. Nifedipine is a calcium channel blocker approved for the prophylaxis of chronic stable angina pectoris and hypertension [77]. In obstetrics it is used for the treatment of acute hypertension, and as a tocolytic in preterm labour [78]. Betamethasone is a corticosteroid approved for suppression of inflammatory and allergic disorders; and congenital adrenal hyperplasia [79]. There has been evidence that supports two doses of 12 mg given 24 h apart antenatally to women at risk of preterm delivery to prevent neonatal respiratory distress, and for diagnosis of Crushing’s syndrome [79]. Misoprostol is a synthetic prostaglandin E1-analogue indicated for the healing of duodenal ulcers and gastric ulcers including those induced by non-steroidal anti-inflammatory drugs (NSAIDs) in arthritic patients at risk, whilst continuing their NSAID therapy [80]. In addition, misoprostol can be used for the prophylaxis of NSAID-induced ulcers [80]. Clinicians use it on an off-label basis for termination of pregnancy (TOP), induction of labour, and cervical ripening before TOP [80, 81]. Aspirin is approved for relief of mild to moderate pain, pyrexia; prophylaxis of platelet aggregation; treatment of rheumatic fever, and acute and chronic inflammatory disorders [30]. Aspirin is used off-label for recurrent miscarriage and thrombophilia [82]. The aspirin’s ability to cause vasodilatation of the uterine arteries, allows for it to be used prophylactically for women at risk of having preterm preeclampsia [83].

Another population that often benefits from OLDU is the geriatrics [84]. In this group of patients, the effects of drugs are affected by age-related changes in pharmacokinetics, pharmacodynamics, and homeostasis, making them more susceptible to the side effects of drugs. Evidence shows that the use of drugs to support the effect is particularly important [73]. In elderly care, prescribers should consider costs and choose the drug with the highest potential for success. The use of unapproved, non-indications of drugs can be safely carried out if the accepted practices are followed [73]. However, it is advisable to notify the patient (or caregiver) that the drug is being prescribed without approval to avoid liability for later problems. This provides a clear information about potential benefits, potential problems, and alternative treatment options.

## Disadvantages

### Insufficient evidence

In some cases, OLDU is practiced in the absence of appropriate scientific data. For example, paclitaxel was originally approved for the treatment of ovarian cancer, but after published reports on breast cancer showed its effectiveness, it was used in the treatment of breast cancer years before regulatory approval for this indication [85]. The OLDU matter in India garnered significant media attention in 2003 when it was discovered that letrozole, an anti-breast cancer medication, was being promoted for infertility without regulatory approval [28]. This led the DCGI to initiate an investigation into media reports that several pharmaceutical companies were endorsing letrozole for infertility treatments without valid authorization [28]. In response to the controversy, the DCGI requested the Indian Medical Association (IMA) to prepare a report on the attributes of OLDU [28]. The IMA’s report recommended that Indian doctors should be permitted to prescribe off-label medications if supported by scientific evidence and a medical basis. Despite this recommendation, no formal regulations regarding off-label prescriptions have been established in India [28, 29].

The controversy regarding the diet ingredient fen-phen highlights the hazards of off-label prescribing. "Fen-phen" was a combination of two drugs used to lose weight: fenfluramine (or the closely related dexfenfluramine) and phentermine. The FDA has approved the short-term use of phentermine, fenfluramine, and dexfenfluramine as the appetite suppressant "anorectic agents" [62]. In the 1990s, it was reported that the "fen-phen" combination resulted in significant reduction in weight. As a result, the number of "fen-phen" prescriptions increased significantly.

Fenfluramine, dexfenfluramine, and phentermine were all FDA-approved drugs, but the combination of "fen-phen" was not individually approved by the FDA, hence, its prescription was termed off-label [86]. Subsequently, in the late 1990s, approximately 24 women taking fen-phen were reported to develop heart valve disease (HVD). Additional small studies have shown that HVD is common in women taking fen-phen [87]. Further examination of the data confirmed the association of HVD with fenfluramine and dexfenfluramine, and thus they were discontinued. Critics of off-label use claimed that the combination of fenfluramine and phentermine had never been approved by the regulatory and was therefore regarded as off-label use, which led to health hazards [86].

Catastrophic events such as the thalidomide-related phocomelia epidemic were painful lessons for healthcare professionals globally. Since then, drug safety concerns have contributed to the development and use of clinical and epidemiological methods for assessing the benefits and potential risks of all types of therapeutic interventions, whether pharmacological or not [87].

### Clinicians’ non-disclosure

Discussions on off-label use of medicines are primarily conducted by consultants per specialty and clinical pharmacologists. Patients and patient caregivers are rarely involved in the most technical issue(s) [4]. This is unsatisfactory from an ethical point of view. The affected persons should know drug approval status including many other things [30]. Patient knowledge and decisions regarding off-label treatment are especially important for alternative therapies where one drug is not approved, and the other drug is approved [4]. But both alternatives are deemed equivalent from a medical point of view. From an ethical point of view, the ethical requirement of ‘informed consent’ means that patients should be informed of any pharmaceutical product used on an off-label basis [88]. Therefore, clinicians are at risk of lawsuits due to the adverse effects associated with when medicines are used on an off-label basis. A study involving 46,021 patients at primary care clinics in Québec, Canada, found that the rate of adverse drug events was 44% higher for OLDU compared to on-label use [89]. This finding held even after accounting for factors such as drug class, drug age, patient age and comorbidities, polypharmacy, and continuity of care [89]. The legal theories used include the unregulated use of research drugs, the failure to obtain informed consent for OLDU, and medical malpractice [4].

## Conclusion

It is not illegal for medical practitioners to use medicines on an off-label basis. However, it is, illegal for pharmaceutical companies to promote drugs and their health products for off-label use. According to the FDA, approved medicines and/or devices advertised for unauthorised use have been incorrectly labeled or tampered with. Thus, discourages the distribution and/or advertisement by any pharmaceutical companies as this can be legally deemed misleading and non-factual. Some may believe that pharmaceutical companies provide transparency with regard to treatment choice as, opposed to the opinion that it poses a significant risk to public health and well-being.

The OLDU can be compared to a double-edged sword that can be utilised fruitfully by medical practitioners. However, if there is uncertain about the scientific relevance of off-label use it can expose the patient to unauthorised experiments, unknown health risks and exposure to ineffective medicines. Therefore, there is an urgent need for guidance, in South Africa, on regulatory framework for OLDU. Furthermore, timeous processing and approval of additional indications of medicines, where there is sound scientific evidence to do so, is needed. This will alleviate a heavy burden, and possible risks that medical practitioners inherently-carry when they compassionately use medicines on an off-label basis.

## Data Availability

Not applicable for this review.

## Abbreviations

SAHPRA: South African Health Products Regulatory Authority
FDA: Food and Drug Administration
USA: United States of America
TGA: Therapeutics Good Administration
OLDU: Off-label drug use
HIV: Human immunodeficiency virus
HAART: Highly active antiretroviral therapy
IV: Intravenous
NSAID: Non-steroidal anti-inflammatory drugs
TOP: Termination of pregnancy
HVD: Heart valve disease
IMA: Indian Medical Association
DCGI: Drug Controller General of India
US: United States
UK: United Kingdom
